# Occupational biohazard exposure during patient waste management: PPE use, reporting practices, and systemic barriers in nursing care

**DOI:** 10.1016/j.infpip.2026.100586

**Published:** 2026-08-26

**Authors:** Debra D. Harris, Rodney X. Sturdivant

**Affiliations:** a Human Sciences and Design, Baylor University, One Bear Place #97346, Waco, TX, USA; b Statistical Science, Baylor University, Waco, TX, USA

**Keywords:** Biohazards, Personal protective equipment, Occupational exposure, Infection control, Waste management, Safety culture

## Abstract

**Background:**

Nurses face occupational exposure risks during patient waste management, yet this aspect of nursing practice remains understudied. This study examined personal protective equipment (PPE) use during patient waste management and explored the behavioural, organizational, and environmental factors influencing adherence to infection prevention practices.

**Methods:**

A mixed-methods national survey of nurses (*N* = 683) was conducted. Quantitative data were analysed using Kruskal–Wallis and χ^2^ tests, while open-ended responses underwent thematic analysis. Associations between PPE-related practices and demographic and organizational characteristics were examined to identify patterns in protection, reporting, and risk perception.

**Results:**

Glove use was nearly universal; however, face masks, gowns, eye protection, face shields, and respirators were used less consistently during waste-management activities. Quantitative and qualitative findings identified barriers, including limited PPE accessibility, time constraints, discomfort, and reliance on perceived rather than actual risk. Nurses frequently described splash exposures as routine, and many reported greater willingness to report needlestick injuries than splash or spill exposures. Infrastructure limitations, including poorly located utility rooms and limited mitigation devices, further contributed to inconsistent protective practices.

**Conclusions:**

Patient waste management represents an underrecognized source of occupational biohazard exposure in nursing care. PPE adherence is influenced by interacting behavioural, cultural, organizational, and environmental factors. Findings highlight the importance of addressing exposure desensitization, improving reporting practices, strengthening access to PPE and mitigation technologies, and fostering a safety culture that supports consistent adherence to infection prevention protocols.

## Introduction

Personal protective equipment (PPE) is essential in hospital settings to safeguard healthcare workers from infectious diseases. The proper use of PPE, including gloves, gowns, face masks, face shields, and respirators, is critical for infection control and preventing occupational exposure [1]. Nurses face significant risks of exposure to infectious agents during waste management, particularly when handling urine, faeces, vomit, or other body fluids. These exposures can occur through (1) splashes or spills that contact the eyes, nose, mouth, or broken skin; (2) aerosolization of pathogens during flushing or manual cleaning of bedpans and commodes; and 3) cross-contamination via surfaces, equipment, or improperly disposed waste [1].

Common pathogens of concern include *Clostridioides difficile* (*C. diff*) [[2], [3], [4]], *Norovirus* [[5], [6], [7]], *Hepatitis A, B, and C* (HAV; HBV; HCV) [8,9], and other enteric or blood-borne viruses [10,11]. Inadequate PPE use or inconsistent hygiene practices increase the likelihood of occupational exposure [[12], [13], [14], [15]]. Lee *et al.* [12] found that most exposures occurred in patient units, emergency rooms, and operating rooms, primarily through percutaneous injuries. Despite repeated exposures to HBV, HCV, and human immunodeficiency virus (HIV), there was a low transmission rate, but the study underscores the importance of preventive measures. A study focused on occupational exposure to respiratory pathogens during aerosol-generating procedures found that inadequate use of PPE, such as N95 respirators, significantly increased the risk of transmission of diseases like influenza and tuberculosis [13].

Adherence to PPE protocols among nurses is essential for infection control and the safety of both healthcare workers and patients. However, compliance often varies significantly due to a combination of institutional, psychological, and logistical factors. Numerous studies have explored the reasons behind fluctuating PPE use and have highlighted key areas for improvement [[16], [17], [18]].

Institutional policies and support play a critical role in promoting adherence. Chughtai *et al.* [19] found that proper PPE usage is closely tied to the presence of clear protocols, adequate training, and consistent availability of protective equipment. Similarly, Verbeek *et al.* [20] demonstrated that hospitals with structured, hands-on training programmes and accessible guidelines achieved higher compliance rates among nursing staff. Conversely, several barriers contribute to reduced adherence. MacIntyre and Chughtai [21] emphasized that the discomfort associated with PPE, the time-consuming nature of donning and doffing procedures, and the complexity of using multiple protective layers can hinder consistent usage. These physical and procedural challenges are further compounded by supply shortages, especially during pandemics like coronavirus disease 2019 (COVID-19), which have forced some nurses to reuse PPE or work with insufficient protection [22].

Additional studies point to high workload and limited access to PPE as significant deterrents to consistent compliance [23]. Psychological factors also influence behaviour: while fear of infection can motivate adherence, overconfidence in personal immunity may reduce it [20]. The prevailing workplace culture and peer practices also exert a strong influence on individual compliance, as noted by Khoshakhlagh *et al.* [24], suggesting that collective norms and leadership behaviour shape PPE practices. Moreover, PPE-related discomfort extends beyond the physical. While prolonged use of face masks can cause skin irritation, it can also hinder verbal and non-verbal communication, particularly in high-stress clinical environments, which may compromise patient care quality and safety [25]. These challenges are magnified during tasks involving waste management, where improper or inconsistent PPE use can heighten the risk of exposure to infectious materials [1].

In response to these challenges, several strategies have been proposed to enhance PPE adherence. Educational and training initiatives remain the most consistently recommended, with studies showing that these programmes improve both knowledge and compliance [26]. Reinforcing training with monitoring systems and real-time feedback can also strengthen adherence [16]. At the design level, improving the ergonomics and comfort of PPE has the potential to reduce physical barriers to use [17]. Importantly, tailoring PPE protocols to include guidance on high-risk activities such as body waste disposal can help address overlooked areas of exposure and further increase compliance [1].

This study evaluates nurses’ adherence to PPE protocols during patient waste management by identifying the systemic, behavioural, and environmental factors that influence compliance and assessing associated occupational risks. Unlike prior studies that have examined PPE use broadly across healthcare settings, this study focuses specifically on patient waste management as an underexplored source of occupational biohazard exposure and integrates quantitative and qualitative data to better understand the factors that shape protective behaviours and reporting practices.

## Methods

### Subject selection and recruitment

The study targeted nurses in roles requiring an associate, bachelor’s, or advanced degree. Participants were recruited via a nursing database and a flyer distributed at the 2024 ANCC National Magnet Conference. Each received a link and Quick Response (QR) code directing them to the consent page; access to the survey followed consent. All participants were 18 years or older, and no vulnerable populations were included. Notice of exemption (IRB Reference #: 2239496) from Institutional Review Board (IRB) review was granted on 16^th^ September 2024.

### Survey tool

A 13-item survey focused on PPE was developed for this study, combining quantitative and qualitative elements. Two open-ended questions captured qualitative insights, while the final four items collected demographic data used as dependent variables. No personally identifiable information was gathered, and participation was voluntary to reduce bias. The survey, administered in English via Qualtrics [27], was conducted from 15^th^ October 2024 through the end of 2024.

Incomplete surveys were included, with unanswered items noted. Data were removed upon participant withdrawal. Qualitative responses were categorized based on prevalence and aligned with quantitative findings. All responses were anonymous, and the research team did not collect or have access to any personally identifiable information. Participants were selected from a professional organization’s roster using role-based criteria. The study received IRB exemption on 16^th^ September 2024. All statistical analysis was performed using R [28].

### Instrument development and validation

**Content validity.** Survey items were developed following a review of the literature on PPE use, occupational exposure, infection prevention, and waste-management practices. An expert panel reviewed the instrument for clarity, relevance, and content coverage to establish content validity and ensure alignment with current PPE and infection-control guidelines.

**Reliability.** Internal consistency of PPE-use items was assessed using Cronbach’s α and Kuder–Richardson 20 (KR-20). Cronbach’s α demonstrated acceptable reliability for the PPE-use scale (α = 0.79; *N* = 632), increasing to α = 0.83 when glove use was excluded because of near-ceiling responses. For dichotomized PPE-use items, KR-20 was 0.75 for the six-item composite and 0.78 when gloves were excluded, indicating acceptable internal consistency. The two-item protection-behaviour scale yielded an interitem correlation of 0.28 and Cronbach’s α of 0.43, which is expected for short scales.

**Construct validity (EFA).** Exploratory factor analysis supported the construct validity of the instrument. A two-factor solution (eigenvalues = 3.01 and 1.01, respectively) explained approximately 67% of the variance. Factor 1 represented non-glove PPE use (face mask, shield, gown, respirator, and goggles), whereas factor 2 represented glove use. This structure was retained based on interpretability and parsimony.

### Quantitative and qualitative analyses

**Demographics.** Survey items on participant characteristics were summarized, with missing responses noted. A total of 140 entries were excluded due to complete non-response (consent given but no questions answered), resulting in a final sample of *N* = 683. Missing data ranged from 0.1% to 8.2%, with all item-level sample sizes exceeding 600. Little’s Missing Completely at Random (MCAR) test (*P* = 0.28) supported the assumption that data were missing completely at random [29]. Thus, complete case analysis was used, minimizing bias given the low proportion of missing data.

**Quantitative analysis.** Research questions (Table I) were analysed alongside demographic data. Questions 1–3 used ordinal responses and were assessed using the Kruskal–Wallis test [30] to detect differences across demographic groups. A significant result (*P* < 0.05) indicated differing medians; post hoc comparisons were conducted using the Dunn test [31] with Holm adjustment [32] to control for multiple comparisons (Table II).

**Table I:** List of survey questions about PPE

1. Repeated exposures to body waste spills and/or splashes desensitize healthcare workers to health risks. 2. I protect myself with appropriate PPE when disposing of sharps containers/objects and/or manually cleaning bedpans and commode pails. 3. What type of personal protection equipment (PPE) do you wear while providing patient care related to patient waste? 4. Wearing PPE while providing patient care related to patient body waste is difficult because: these items are not available to use in my unit, not always available to use in my unit, not conveniently available to use in my unit, or other. 5. Reasons for not using PPE: it takes too much time to get the PPE and put it on, PPE is uncomfortable to wear, PPE hinders my ability to perform tasks, or it feels wasteful to use PPE that gets thrown away. 6. If PPE is available and accessible, why do you think PPE is not used for protection during patient care and cleaning of bedpans and commode pails? 7. Manual cleaning of bedpans and commode pails is performed multiple times a day for each patient. How do you perceive this occupational exposure to patient waste via spills and splashes during manual cleaning of bedpans and commode pails? 8. Do you feel more comfortable reporting a needlestick injury than a splash and spill on your eyes, nose, and mouth during the process of patient care or manual cleaning of bedpans and commode pails? 9. Which of the following mitigation mechanisms and devices has your hospital implemented? Choices included: macerators, bedpan liners, splash screens, bedpan, and commode pail liners, or none.

**Table II:** Summary of participant demographics (*N* = 683)

	No. of participants	Sample %
**Professional designation**		
Nurse practitioner (NP)	4	0.6
Registered nurse (RN)	586	86
Licensed practical nurse (LPN)	10	1.5
Certified nursing assistant (CNA)	6	0.9
Advanced practice registered nurse (APRN)	8	1.2
Clinical nurse specialist (CNS)	9	1.3
Certified nurse educator (CNE)	7	1.0
Infection preventionist (IP)	27	4.0
Other	24	3.5
Missing	2	
**Age (yrs)**		
18–20	1	0.1
21–29	74	11
30–39	160	24
40–49	187	28
50–59	179	26
60+	78	11
Missing	4	
**Experience (yrs)**		
0–5	63	9.2
6–10	99	15
11–15	111	16
16–20	95	14
21+	314	46
Missing	1	

*N* (%).

Remaining questions were categorical and analysed using χ^2^ tests. Significant associations (*P* < 0.05) between demographics and responses were reported. Only significant results were presented due to the size of contingency tables.

**Qualitative analysis.** Two open-ended questions addressed PPE use: (1) what would increase your use of PPE for eye, nose, and mouth protection during patient care and waste management and (2) what are your comments on issues with waste management. Responses were analysed using thematic analysis with supplemental quantitative coding. An inductive approach allowed factors to emerge from the data. Responses were reviewed for relevance, and key patterns were identified. Frequency counts quantified factor prevalence. To ensure reliability, two researchers independently coded responses and cross-verified factors. Findings were contextualized with literature on occupational health, safety culture, and PPE compliance. The final framework highlighted barriers and facilitators to PPE use, informing policy and best practices.

## Results

### Demographics

The final sample included 683 participants who answered at least one survey question. Overall, 83% of respondents completed the survey. Most were registered nurses (86%) and between 30 and 60 years old (78%). Nearly half (46%) had over 20 years of experience, with fewer in the 0–5 year range.

Table III summarizes facility types and accreditation status. Most respondents (70%) worked in community hospitals. Half reported working at Magnet or Pathway-designated facilities.

**Table III:** Summary of facility types (*N* = 683)

Facility Accreditations	No. of participants	Sample %
**Type of facility**		
Community	471	70
University	73	11
Government	20	3.0
Other	113	17
Missing	6	
**Accreditation/certification**		
Magnet	238	35
Pathway to Excellence	103	15
Neither	301	45
Other	30	5.0
Missing	11	

*N* (%).

### Quantitative findings: PPE use, risk perceptions, and reporting practices

Structured survey responses (excluding open-ended items) revealed key trends in PPE use and perceptions among nurses. Most (69%) agreed that repeated exposure to patient waste leads to desensitization of PPE risks, defined as the increased likelihood of splash exposure, contamination, or pathogen transmission when appropriate protective equipment is not used.

Still, 88% reported ‘always’ or ‘most of the time’ using PPE when handling sharps or cleaning bedpans and commode pails. PPE use varied substantially by equipment type. While glove use was nearly universal, utilization of face masks, gowns, face shields, eyewear, and respirators was considerably lower, indicating inconsistent adherence to protective measures beyond routine glove use.

The most cited barrier to PPE use was the time required to don and doff. Other challenges included inconvenience, limited availability, discomfort, perceived wastefulness, and low perceived risk. Regarding mitigation strategies, 67% reported none in place, while 24% noted the use of liners for bedpans and commode pails.

Demographic and organizational variables – age, experience, role, facility type, and hospital designation – were analysed based on prior research linking them to PPE knowledge, reporting behaviour, mitigation protocols, and satisfaction. These factors helped assess how individual and institutional contexts shape risk perception and compliance.

***Age.*** Significant age-related differences were identified for PPE use while cleaning bedpans (*P* = 0.018) and for the use of gloves (*P* = 0.038), face masks (*P* = 0.049), gowns (*P* = 0.001), and respirators (*P* = 0.027) (Table IV). No significant age-group differences were observed in perceptions that repeated exposure to patient waste desensitizes healthcare workers to occupational risk. Across all age groups, PPE use during sharps handling was consistently high, whereas goggle use remained low. Subsequent post hoc analyses identified the specific age-group differences contributing to these findings.

**Table IV:** PPE use, reporting preferences, and waste management practices by age

Variable	<29 *N* = 75	30–39 *N* = 160	40–49 *N* = 187	50–59 *N* =179	60+ *N* = 78
**PPE Use by Mitigation Type**					
Desensitize	3.61 (1.44)	3.59 (1.44)	3.65 (1.36)	3.63 (1.39)	3.26 (1.45)
Protect-Sharp	4.45 (0.74)	4.45 (0.91)	4.43 (1.04)	4.39 (0.98)	4.34 (1.05)
Protect-Bedpan^a^	4.45 (1.02)	4.26 (1.24)	4.60 (0.90)	4.54 (1.02)	4.57 (0.96)
Gloves^a^	4.76 (0.46)	4.83 (0.52)	4.89 (0.37)	4.86 (0.40)	4.87 (0.55)
Face mask^a^	2.60 (1.09)	2.61 (1.22)	2.43 (1.23)	2.85 (1.37)	2.61 (1.33)
Shield	1.60 (0.79)	1.81 (0.96)	1.88 (1.07)	2.12 (1.25)	1.99 (1.25)
Gown^a^	2.36 (0.97)	2.39 (1.06)	2.50 (1.17)	2.92 (1.29)	2.78 (1.35)
Respirator^a^	1.77 (0.89)	1.53 (0.84)	1.52 (0.90)	1.71 (1.10)	1.43 (0.67)
Goggles	1.65 (0.94)	1.68 (0.96)	1.76 (0.99)	1.99 (1.23)	1.93 (1.17)
**Reasons for not using PPE** (%)					
Too much time	61 (42)	88 (36)	96 (33)	89 (34)	38 (33)
Uncomfortable	21 (14)	33 (13)	48 (16)	46 (18)	18 (16)
Hinders tasks	24 (17)	44 (18)	59 (20)	40 (15)	19 (16)
Wasteful	32 (22)	47 (19)	44 (15)	37 (14)	15 (13)
Other	7 (4.8)	35 (14)	45 (15)	47 (18)	26 (22)
**Preference for reporting needlestick over splash/spill exposures** (%)					
Yes	58 (73)	108 (65)	102 (53)	107 (59)	35 (44)
No	18 (23)	45 (27)	85 (44)	70 (38)	39 (49)
Less punitive	2 (2.5)	11 (6.7)	5 (2.6)	4 (2.2)	4 (5.0)
Do not report	1 (1.3)	1 (0.6)	1 (0.5)	1 (0.5)	2 (2.5)
**Mitigation mechanisms and devices implemented by hospitals** (%)					
Macerators	0 (0)	0 (0)	0 (0)	2 (1.1)	0 (0)
Bedpan liners	1 (1.4)	12 (7.5)	4 (2.2)	2 (1.1)	3 (4.1)
Splash screens	0 (0)	7 (4.4)	11 (6.1)	12 (6.9)	6 (8.1)
Bedpan/commode pail liners	22 (30)	31 (19)	44 (25)	49 (28)	13 (18)
None	50 (68)	109 (69)	120 (67)	110 (63)	52 (70)

PPE, personal protective equipment.

**Notes:** Values are presented as mean (standard deviation [SD]) for PPE use and *N* (%) for all other sections.

Overall age-group differences were observed for reasons for not using PPE (Pearson’s χ^2^, *P* = 0.030), preference for reporting needlestick over splash/spill exposures (Pearson’s χ^2^, *P* = 0.001), and mitigation mechanisms and devices implemented by hospitals (Pearson’s χ^2^, *P* = 0.019.

a Indicates a statistically significant age-group difference for PPE use variables (Kruskal–Wallis rank-sum test, *P* < 0.05).

**Figure 1:**
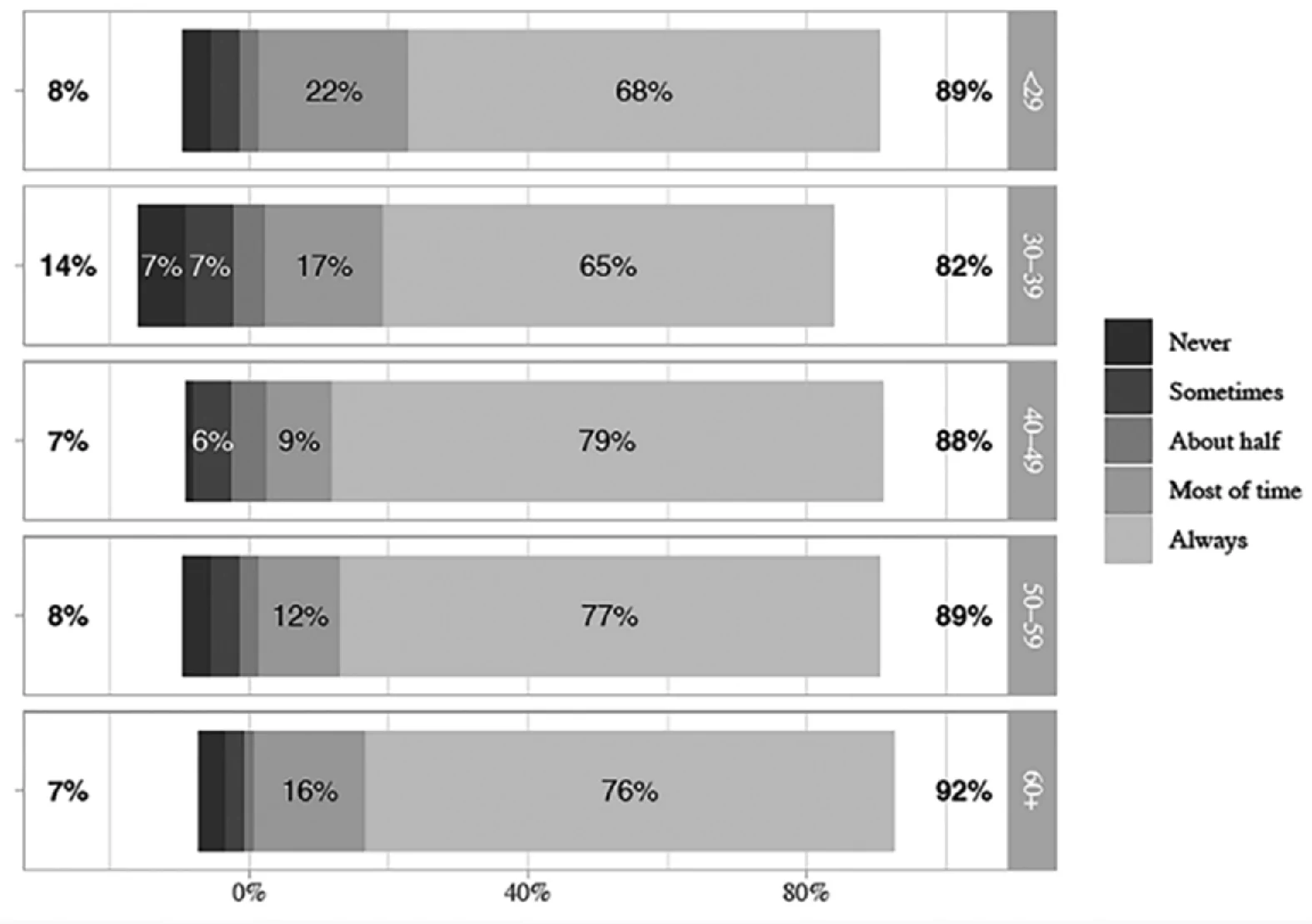
How often nurses protect themselves with PPE when cleaning bedpans or commode pails by age? PPE, personal protective equipment.

PPE use during bedpan cleaning differed by age group (Figure 1). The Dunn test with Holm correction for multiple corrections revealed a significant pairwise difference between the 40–49 and 30–39 age groups (*P* = 0.0243), with the 40–49 group showing the highest mean response and the 30–39 group the lowest. Not significant but following the trend were the pairwise comparisons for age groups 50–59 and 60+ compared with 30–39.

**Figure 2:**
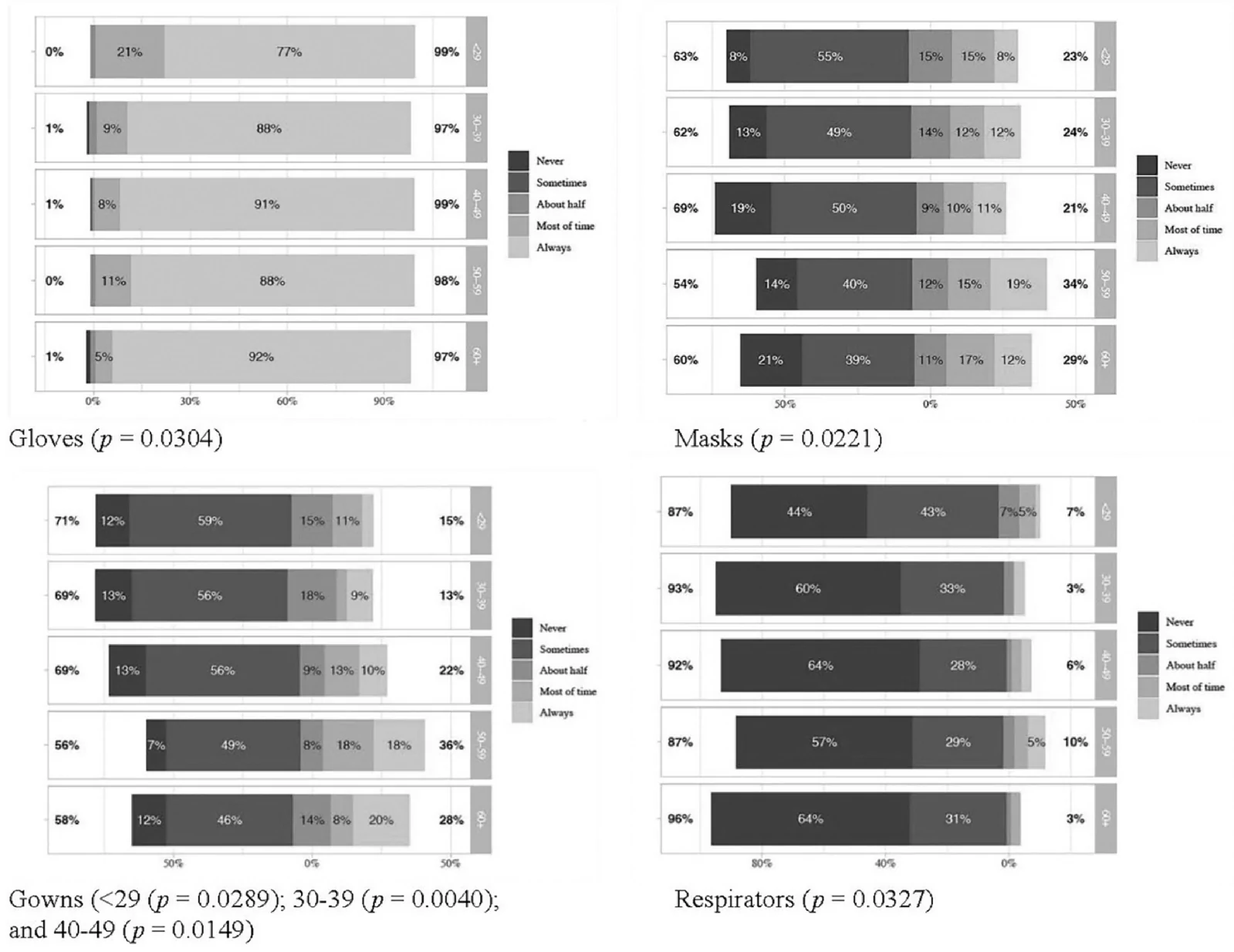
PPE used by nurses while providing patient care and waste management for significance by age. PPE, personal protective equipment.

Age-related differences were observed for several PPE types (Figure 2). Significant age-related differences emerged. Glove use was lower among those under 29 compared with ages 40–49 years (*P* = 0.0304). For face masks, the 40–49 group used them less than the 50–59 group (*P* = 0.0221). Gown use was highest among those aged 50–59 years, significantly more than those under 29 (*P* = 0.0289), 30–39 (*P* = 0.0040), and 40–49 (*P* = 0.0149). Respirator use was highest among the youngest group, significantly more than the 40–49 group (*P* = 0.0327). Collectively, these results suggest that PPE use patterns vary by age, although the direction of the relationship differs by PPE type. Older nurses reported greater use of gowns, whereas younger nurses reported greater respirator use.

Survey participants were asked about reasons for NOT using PPE and had a significant association with age (*P* = 0.03). Content analysis of ‘Other’ revealed recurring sentiments related to availability barriers, time and emergency constraints, low perceived risk, physical discomfort, and attitudinal barriers (desensitization, scepticism).

One question addressed incident reporting and asked, ‘Do you feel more comfortable reporting a needlestick injury than a splash or spill on your eyes, nose, or mouth during patient care or the manual cleaning of bedpans and commode pails?’ Reporting preferences differed by age (*P* = 0.001). Older respondents were progressively less likely to indicate a preference for reporting needlestick injuries over splash or spill exposures, suggesting age-related differences in exposure reporting preferences.

When asked about the type of mitigation mechanisms and devices that the respondents’ hospital has implemented, there was a significant association with age (*P* = 0.019). Most of the nurses, across the age categories responded ‘none,’ with the next highest type of mitigation tool being bedpan/commode pail liners.

***Professional experience.*** Significant differences by professional experience were observed only for PPE use when cleaning bedpans (*P* = 0.024). Although few significant differences were observed, a general trend suggested that more experienced nurses reported greater use of PPE during bedpan cleaning than less experienced nurses.

Significant associations with professional experience were also found for ‘reasons not to use PPE’ (*P* < 0.001), for the question ‘why do you think PPE is not used?’ (*P* = 0.026), and for the question, ‘Are you more comfortable reporting needlestick than splash/spill?’ (*P* = 0.006). As nurses gain experience, they are less likely to report a splash/spill incident (Table V).

**Table V:** PPE use, reporting preferences, and waste management practices by professional experience

Variable	0–5 years *N* = 63	6–10 years *N* = 99	11–15 years *N* = 111	16–20 years *N* = 95	21+ years *N* = 314
**PPE use by mitigation type**					
Desensitize	3.76 (1.36)	3.45 (1.48)	3.79 (1.36)	3.63 (1.35)	3.48 (1.43)
Protect-Sharp	4.39 (0.88)	4.51 (0.71)	4.50 (0.93)	4.37 (1.02)	4.38 (1.04)
Protect-Bedpan^a^	4.19 (1.37)	4.39 (1.10)	4.34 (1.16)	4.60 (0.87)	4.59 (0.94)
Gloves	4.86 (0.35)	4.76 (0.62)	4.86 (0.42)	4.85 (0.41)	4.88 (0.41)
Face mask	2.87 (1.18)	2.55 (1.11)	2.66 (1.30)	2.55 (1.29)	2.60 (1.31)
Shield	1.71 (0.87)	1.68 (0.87)	1.93 (1.09)	1.99 (1.15)	1.97 (1.18)
Gown	2.38 (1.07)	2.38 (0.99)	2.50 (1.10)	2.62 (1.26)	2.74 (1.28)
Respirator	1.79 (0.99)	1.56 (0.84)	1.57 (0.89)	1.61 (0.97)	1.56 (0.94)
Goggles	1.56 (0.74)	1.78 (0.95)	1.67 (1.00)	1.90 (1.17)	1.88 (1.14)
**Reasons for not using PPE** (%)					
Too much time	49 (42)	57 (36)	64 (35)	47 (32)	156 (34)
Uncomfortable	16 (14)	23 (15)	29 (16)	22 (15)	77 (17)
Hinders tasks	19 (16)	31 (20)	34 (19)	35 (23)	69 (15)
Wasteful	27 (23)	32 (20)	37 (20)	15 (10)	64 (14)
Other	6 (5.1)	15 (9.5)	19 (10)	30 (20)	91 (20)
**Perceptions of why PPE is not used** (%)					
Too much time	49 (38)	59 (33)	71 (38)	61 (38)	152 (31)
Burned out	10 (7.8)	12 (6.7)	8 (4.3)	8 (5.0)	18 (3.7)
Low risk	16 (13)	28 (16)	39 (21)	24 (15)	105 (22)
Unrealistic	37 (29)	46 (26)	48 (26)	42 (26)	128 (27)
Creates barrier	14 (11)	23 (13)	17 (9.0)	15 (9.3)	37 (7.7)
Other	2 (1.6)	11 (6.1)	5 (2.7)	11 (6.8)	43 (8.9)
**Preference for reporting needlestick over splash/spill exposures** (%)					
Yes	49 (75)	71 (68)	71 (63)	55 (56)	166 (52)
No	14 (22)	28 (27)	34 (30)	37 (38)	144 (45)
Less punitive	1 (1.5)	4 (3.8)	7 (6.2)	4 (4.1)	10 (3.1)
Do not report	1 (1.5)	1 (1.0)	1 (0.9)	2 (2.0)	1 (0.3)

PPE, personal protective equipment.

Notes: Values are presented as mean (standard deviation [SD]) for PPE use and *N* (%) for all other sections.

a Indicates a statistically significant difference by professional experience for PPE use variables (Kruskal–Wallis rank-sum test, *P* < 0.05). Overall differences by professional experience were observed for reasons for not using PPE (Pearson χ^2^, *P* < 0.001), perceptions of why PPE is not used (*P* = 0.027), and preference for reporting needlestick over splash/spill exposures (*P* < 0.001).

**Figure 3:**
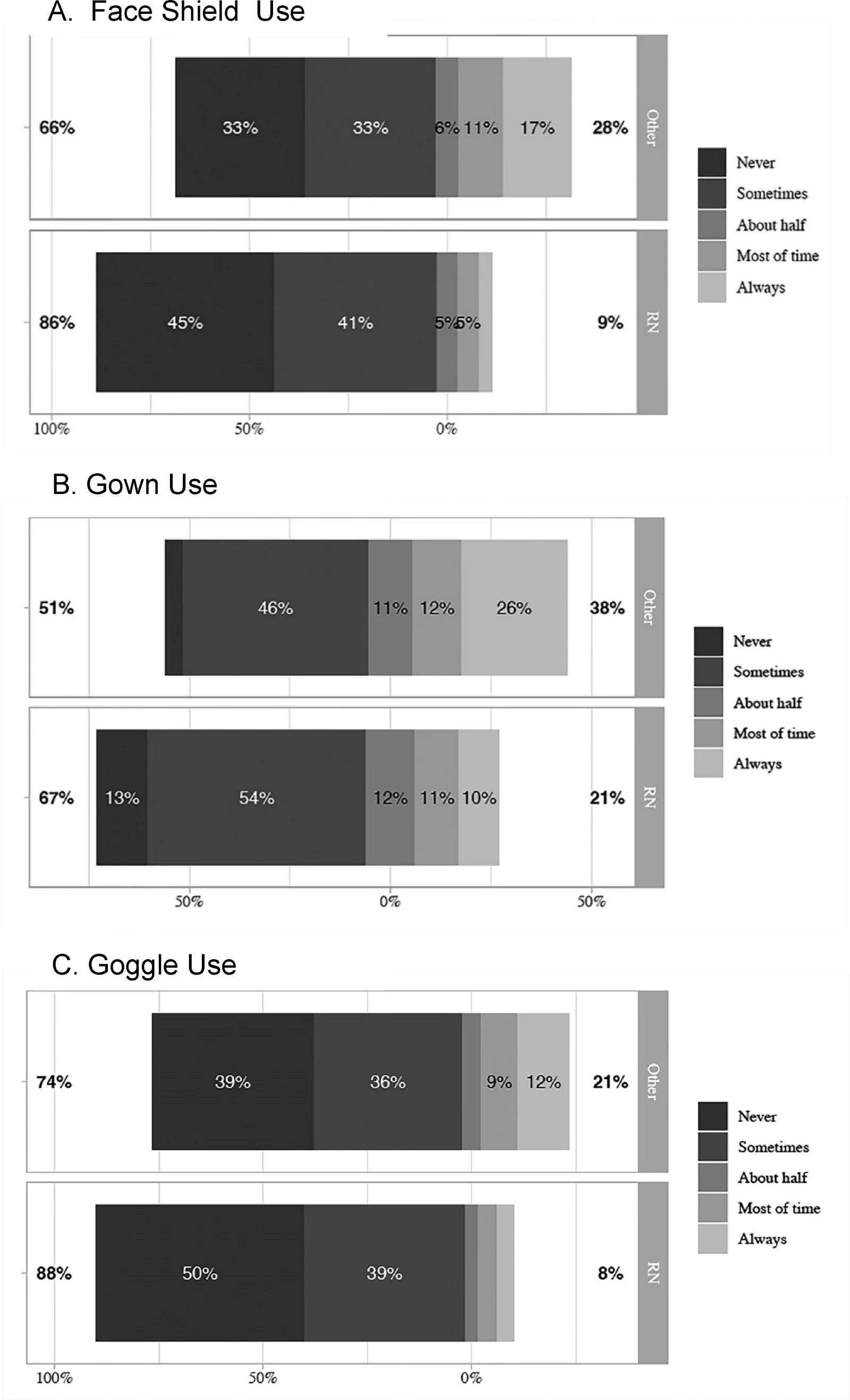
PPE use by clinical role. (A) Face shield use, (B) gown use, and (C) goggle use among registered nurses (RNs) and respondents in other clinical roles. Significant differences were observed for all three PPE types (*P* ≤ 0.05). PPE, personal protective equipment.

***Clinical role.*** Due to the small sample sizes within the non-RN professional designation categories, participants were grouped into two categories: Registered Nurse (RN) and Other. Compared with respondents in other clinical roles, RNs reported significantly lower use of face shields (*P* < 0.001), gowns (*P* < 0.001), and goggles (*P* = 0.004; Figure 3), suggesting differences in PPE practices associated with role responsibilities and exposure perceptions. Additionally, a significant association was observed between clinical role and perceptions of bedpan cleaning. A higher proportion of RNs indicated that this task was either ‘part of the job’ or that they were ‘desensitized’ to it (*P* < 0.001, Table VI).

**Table VI:** PPE use and perceptions of occupational exposure by clinical role

Variable	Other (*N* = 97)	RN (*N* = 586)
**PPE use**		
Desensitize	3.60 (1.25)	3.57 (1.44)
Protect-Sharp	4.36 (1.10)	4.43 (0.94)
Protect-Bedpan	4.45 (1.03)	4.49 (1.05)
Gloves	4.86 (0.52)	4.85 (0.44)
Face mask	2.81 (1.38)	2.59 (1.24)
Face Shield^a^	2.47 (1.48)	1.81 (0.99)
Gown^a^	3.10 (1.35)	2.51 (1.15)
Respirator	1.64 (1.00)	1.58 (0.91)
Goggles^a^	2.20 (1.37)	1.74 (1.00)
**Perception of occupational exposure** (%)		
Part of job	57 (36)	396 (41)
Done for too long	39 (25)	187 (19)
No time to worry	8 (5.0)	83 (8.6)
Will not harm	7 (4.4)	25 (2.6)
Desensitized	28 (18)	228 (24)
Other	20 (13)	49 (5.1)

PPE, personal protective equipment.

Notes: Values are presented as mean (standard deviation [SD]) for PPE use and *N* (%) for perception of occupational exposure.

a Indicates a statistically significant difference by clinical role (Wilcoxon rank-sum test, *P* < 0.05). Significant PPE variables were shield (*P* < 0.001), gown (*P* < 0.001), and goggles (*P* = 0.004). Overall differences in perception of occupational exposure were observed by clinical role (Pearson’s χ^2^, *P* < 0.001).

**Figure 4:**
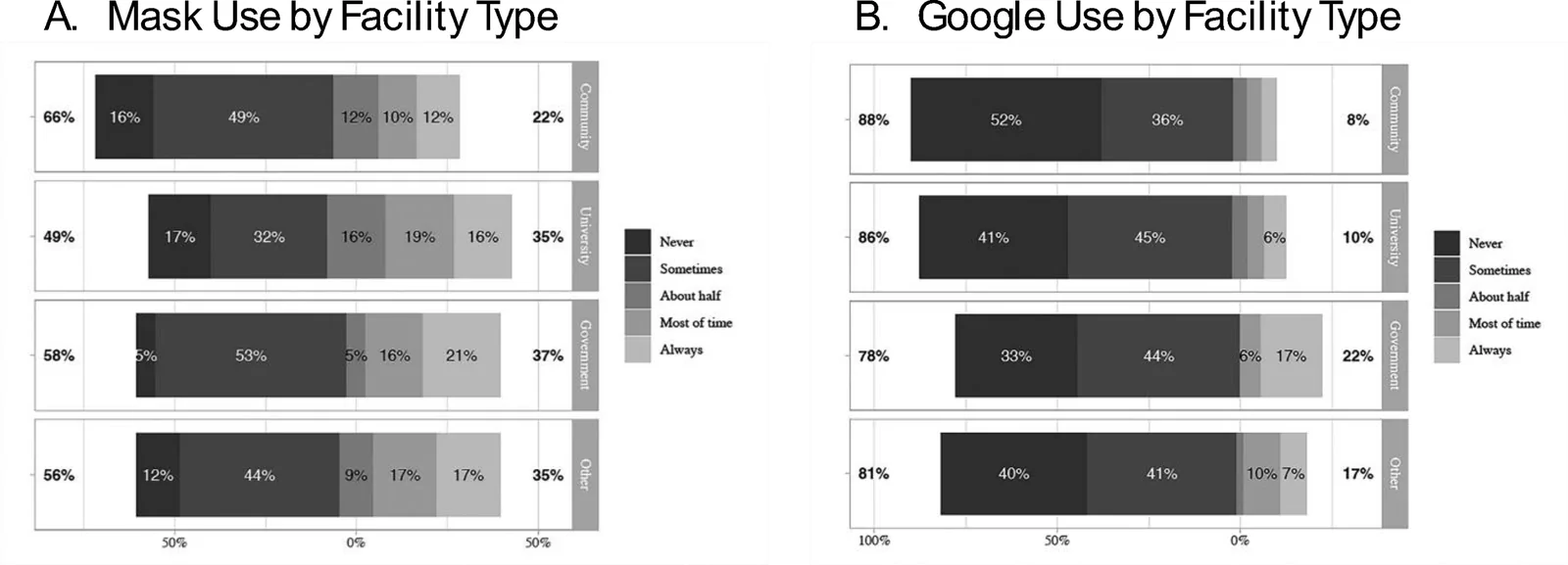
PPE use by facility type. (A) Mask use and (B) goggle use among nurses working in community, university, government, and other healthcare facilities. Significant differences were observed by facility type for both PPE categories. PPE, personal protective equipment.

***Facilitytype.*** Respondents from community hospitals generally reported lower use of face masks and goggles and were more likely to identify accessibility and convenience barriers than respondents from other facility types (Table VII; Figure 4).

**Table VII:** PPE use and barriers to PPE use by facility type

Variable	Community	University	Government	Other
**PPE Use**				
Desensitize	3.61 (1.37)	3.51 (1.53)	3.30 (1.49)	3.58 (1.45)
Protect-Sharp	4.46 (0.91)	4.25 (1.14)	4.30 (1.22)	4.38 (1.00)
Protect-Bedpan	4.48 (1.04)	4.38 (1.15)	4.68 (0.82)	4.51 (1.03)
Gloves	4.86 (0.44)	4.73 (0.61)	4.85 (0.37)	4.88 (0.37)
Face mask^a^	2.52 (1.22)	2.84 (1.36)	2.95 (1.35)	2.84 (1.33)
Face Shield	1.83 (1.03)	1.91 (1.13)	2.06 (1.26)	2.17 (1.28)
Gown	2.56 (1.17)	2.76 (1.30)	3.10 (1.29)	2.59 (1.21)
Respirator	1.57 (0.88)	1.58 (0.90)	1.44 (0.78)	1.71 (1.12)
Goggles^a^	1.71 (1.00)	1.90 (1.07)	2.28 (1.45)	2.04 (1.22)
**Reasons PPE is difficult to wear during patient care** (%)				
Not available	17 (3.5)	4 (5.9)	0 (0)	5 (4.1)
Not always available	85 (18)	8 (12)	3 (15)	22 (18)
Not convenient	218 (45)	25 (37)	4 (20)	37 (31)
Other	164 (34)	31 (46)	13 (65)	57 (47)

PPE, personal protective equipment.

Notes: Values are presented as mean (standard deviation [SD]) for PPE use and *N* (%) for reasons PPE is difficult to wear during patient care.

a Indicates a statistically significant difference by facility type for PPE use variables (Kruskal–Wallis rank-sum test, *P* < 0.05). Significant PPE variables were Face mask (*P* = 0.039) and goggles (*P* = 0.012). Overall differences by facility type were observed for reasons PPE is difficult to wear during patient care (Pearson’s χ^2^, *P* = 0.017).

***Hospitaldesignations.*** The only statistically significant difference by hospital designation was observed for glove use (*P* = 0.005, Table VII). Magnet facility respondents used gloves less often than Pathway to Excellence facilities, facilities that had neither Magnet nor Pathway to Excellence designations, or had another designation not listed in Table VII. The other types of PPE (face mask, shield, gown, respirator, and goggles) were less likely to be used and not significantly different (Table VIII).

**Table VIII:** Use of PPE by hospital designations

Variable	Magnet *N* =238	Pathway *N* =103	Neither *N* =301	Other *N* =30
Desensitize	3.63 (1.47)	3.49 (1.39)	3.58 (1.37)	3.33 (1.44)
Protect-Sharp	4.41 (0.85)	4.40 (1.10)	4.41 (0.99)	4.47 (1.17)
Protect-Bedpan	4.42 (1.05)	4.58 (0.97)	4.49 (1.06)	4.47 (1.14)
Gloves^a^	4.77 (0.56)	4.91 (0.32)	4.88 (0.39)	4.93 (0.37)
Face mask	2.76 (1.32)	2.68 (1.33)	2.49 (1.19)	2.50 (1.25)
Shield	1.89 (1.04)	2.22 (1.37)	1.83 (1.05)	1.72 (0.96)
Gown	2.65 (1.18)	2.74 (1.32)	2.51 (1.15)	2.60 (1.28)
Respirator	1.69 (0.99)	1.63 (0.98)	1.52 (0.86)	1.48 (0.83)
Goggles	1.92 (1.13)	1.85 (1.15)	1.72 (1.00)	1.76 (0.99)

PPE, personal protective equipment.

Notes: Values are presented as mean (SD).

a Indicates a statistically significant difference by hospital designation (Kruskal–Wallis rank-sum test, *P* < 0.05).

### Qualitative findings: issues in waste management observed by nurses

***Key factors.*** Open-ended responses revealed challenges in PPE use, infrastructure, occupational exposure, training, and workplace culture.

***PPEcompliancebarriers.*** Despite protocols, PPE use was inconsistent. Contributing factors included post-COVID desensitization, time constraints, inconvenience, and PPE unavailability. Staff often based PPE use on perceived risk, skipping it for non-isolated patients. Visitors also entered isolation rooms without protection, undermining infection control.

***Infrastructureissues.*** Nurses cited a lack of dedicated waste disposal areas, poorly designed clean spaces, and distant utility rooms. Outdated or broken equipment (e.g. bedpans, sprayers) and absence of disposable liners increased exposure risks.

***Occupational exposure.*** Nurses frequently encountered unreported exposure during routine care (e.g. catheter flushing, diaper changes). Limited access to PPE – especially eye protection – led some to buy their own. Exposure was often normalized, reflecting a weak safety culture.

***Workplace culture.*** Many viewed exposures as part of the job. Time pressures encouraged shortcuts, and older nurses were generally more cautious. Stigma and top-down PPE policies without frontline input discouraged adherence.

***Need for Standardization and Training.*** Inconsistent PPE practices and unclear guidelines highlighted the need for standardized protocols and better education. New nurses lacked bedside training, revealing gaps in onboarding and ongoing instructions.

## Discussion

This study examined nurses’ use of PPE during patient waste management and identified interconnected behavioural, organizational, and environmental factors that contribute to occupational biohazard exposure. By integrating survey findings with nurses’ narrative accounts, the results demonstrate that routine waste management is frequently perceived as low risk despite persistent opportunities for splash and aerosol exposure. A unique contribution of this study is its focus on patient waste management as a distinct occupational exposure context and its identification of desensitization and reporting preferences as factors influencing PPE-related decision-making.

A consistent finding across both datasets was the selective use of PPE during waste-management activities. Quantitatively, glove use was nearly universal, whereas face masks, gowns, face shields, and eye protection were used far less frequently. Qualitative responses provided context for these patterns, with nurses frequently citing limited availability, inconvenience, time pressures, and reliance on perceived rather than actual risk when deciding whether to use PPE. Together, these findings suggest that waste-related exposures are often managed through individual risk assessment rather than consistent application of standard precautions. Similar barriers to PPE compliance have been identified in previous studies, including discomfort, accessibility challenges, workload demands, and the tendency for healthcare workers to modify PPE use based on perceived levels of risk rather than established infection-control guidelines [17,[19], [20], [21]]. Consistent with prior research demonstrating the influence of workplace norms and safety culture on protective behaviours [18,24], participants in the present study frequently described making PPE decisions according to situational judgements rather than routine adherence to standard precautions. These findings extend previous work by demonstrating how such barriers and decision-making processes operate in the context of patient waste management, where opportunities for splash and aerosol exposure are common but may be underestimated [1]. Unlike many previous studies that examined PPE compliance broadly across clinical activities [17,[19], [20], [21]], the present findings suggest that routine waste management may represent a distinct context in which standard precautions are inconsistently applied despite recognized exposure risks.

The quantitative findings and narrative comments collectively suggest that desensitization is a key mechanism influencing PPE behaviour during waste management. Nurses frequently described splash exposure as routine and unavoidable, while survey responses indicated broad agreement that repeated exposure reduces sensitivity to occupational risk. This normalization of exposure may explain why protective practices decline during routine tasks despite awareness of infection-control principles. Although previous studies have identified complacency, fatigue, and declining vigilance as contributors to PPE non-compliance [17,20], the present findings suggest that repeated waste-related exposure may represent a specific pathway through which occupational risks become normalized within everyday nursing practice.

The findings further indicate that PPE adherence is shaped by organizational and environmental conditions. Qualitative responses identified poorly located utility rooms, inadequate disposal facilities, outdated equipment, and limited access to mitigation devices as barriers to safe practice. These infrastructure challenges were frequently discussed alongside reports of splash exposure and inconsistent PPE use, suggesting that environmental conditions directly influence how occupational risks are perceived and managed. The quantitative findings support these observations, with many respondents reporting limited availability and accessibility of PPE and mitigation devices. Consistent with previous research linking environmental design, equipment availability, and workplace resources to occupational exposure risk and PPE compliance [22,26], these findings suggest that the physical work environment plays an important role in shaping safety behaviours. Moreover, the integration of survey and narrative data indicates that when exposure risks become embedded in routine workflows, inadequate infrastructure may contribute to the normalization of exposure and reduce adherence to protective practices. While previous research has primarily emphasized PPE availability and accessibility as determinants of compliance [22,26], the present findings highlight the broader influence of workflow design, utility room location, disposal processes, and mitigation technologies on exposure risk and safety behaviour.

Another important finding was the difference in reporting preferences between needlestick injuries and splash or spill exposures. Across demographic groups, respondents indicated greater willingness to report needlestick injuries than mucous membrane exposures. Qualitative comments further suggested that splash incidents are often perceived as routine events rather than reportable occupational exposures. This discrepancy may contribute to underrecognition of the true burden of waste-related exposure events [12,14]. Although underreporting of occupational exposures has been documented previously [12,14], the current findings suggest that reporting behaviours may differ according to exposure type, with splash and spill events appearing particularly vulnerable to normalization and underreporting.

Workplace culture emerged as a critical reinforcing factor. Many nurses described exposure as part of the job, citing time pressure, staffing shortages, and peer norms that discouraged full PPE use during routine care [1,15]. These cultural signals, particularly when modelled by leadership, function to legitimize risk-taking and silence reporting, perpetuating a cycle of exposure normalization. This finding supports previous research demonstrating the influence of organizational culture and peer behaviours on PPE compliance [18,24], while further illustrating how those influences shape responses to routine waste-management activities.

Taken together, the findings demonstrate that PPE use during patient waste management is influenced by multiple interacting factors, including risk perception, workplace culture, infrastructure design, and reporting practices. The convergence of quantitative and qualitative findings suggests that improving safety in waste management requires a systems-based approach that extends beyond individual education and addresses organizational processes and environmental conditions that normalize occupational exposure [16,24]. Importantly, this study contributes to the existing literature by identifying patient waste management as an underrecognized area of occupational biohazard risk and by highlighting desensitization and differential reporting behaviours as mechanisms that may influence PPE adherence and exposure recognition.

### Practice, policy, and design recommendations

The findings suggest several opportunities to improve occupational safety during patient waste management. First, healthcare organizations should strengthen access to PPE and provide targeted education that emphasizes splash and aerosol exposure risks during routine waste-handling activities. Training should specifically address the tendency to rely on perceived risk rather than standard precautions and reinforce the importance of consistent PPE use across all patient care settings.

Second, organizations should promote a safety culture that encourages appropriate PPE use and exposure reporting. Leadership engagement, visible role modelling, and non-punitive reporting systems may help reduce the normalization of splash exposures and improve recognition of occupational risk.

Finally, environmental and engineering controls should complement behavioural interventions. Improving the design of soiled utility rooms, increasing access to mitigation devices such as bedpan liners and splash shields, and implementing automated waste-management technologies may reduce exposure opportunities and lessen reliance on individual compliance alone.

***Limitations.*** This study has several limitations. First, findings were based on self-reported data and may be subject to recall and social desirability bias. Second, participation was voluntary and recruitment occurred through a professional organization and conference outreach, creating the potential for sampling and non-response bias. Although the sample included nurses from diverse backgrounds and healthcare settings, the findings may not be fully generalizable to all nursing populations or institutions. Additionally, the cross-sectional design limits causal inference, and PPE practices were not directly observed. Because the analyses were exploratory in nature, the study relied primarily on bivariate statistical methods and did not include multi-variable modelling or effect-size measures, limiting assessment of the independent contribution and practical significance of observed relationships. Future studies incorporating observational, longitudinal, and multi-variable approaches may provide a more comprehensive understanding of occupational exposure risks and PPE adherence during patient waste management.

This study directly evaluated nurses’ adherence to PPE protocols during patient waste management and identified substantial gaps beyond routine glove use. Although glove use was nearly universal, the use of gowns, face masks, eye protection, and face shields during waste-related tasks was considerably lower despite the potential for splash and aerosol exposure.

The findings indicate that PPE compliance is influenced by a combination of behavioural, organizational, and environmental factors. Repeated exposure to patient waste was associated with perceived desensitization to risk, while splash and spill exposures were reported less readily than needlestick injuries. Limited PPE accessibility, time constraints, workplace norms, and infrastructure challenges further contributed to inconsistent protective practices.

Overall, patient waste management represents an underrecognized area of occupational biohazard risk in nursing care. Improving worker protection will require healthcare organizations to strengthen safety culture, enhance access to appropriate PPE, support exposure reporting, and address environmental barriers that hinder consistent adherence to infection prevention practices.

## CRediT authorship contribution statement

**Debra D. Harris:** Writing – review & editing, Writing – original draft, Visualization, Validation, Supervision, Resources, Project administration, Methodology, Investigation, Funding acquisition, Conceptualization. **Rodney X. Sturdivant:** Writing – review & editing, Writing – original draft, Visualization, Validation, Resources, Methodology, Funding acquisition, Formal analysis, Data curation, Conceptualization.

## Ethical approval

The Baylor University Institutional Review Board (IRB) determined that this research was exempt from full ethics review under federal regulation 45 CFR 46.104(d) (2): Research involving the use of educational tests, survey procedures, interview procedures, or observation of public behaviour. All participants were informed of the purpose of the study and participation was voluntary.

## Funding sources

This work was supported by Cleanis, Inc. The sponsor provided financial support for the study but had no role in the study design, data collection, data analysis, data interpretation, manuscript preparation, or the decision to submit the manuscript for publication.

## Conflict of interest statement

The authors declare no conflicts of interest. Although this research was funded by Cleanis, Inc., the sponsor had no role in the study design, data collection, data analysis, interpretation of findings, manuscript preparation, or publication decisions.
